# Supplementary material for: Resolving the full spectrum of human genome variation using Linked-Reads
Source: Genome Res. 2019 Apr;29(4):635–45. doi: 10.1101/gr.234443.118 (PMC6442396; doi:10.1101/gr.234443.118)
Supplement: Supplemental Material [file supp_gr.234443.118_Supplemental_longranger-2.2.zip › longranger-2.2.tar.bz2!/lr2.2-public/lib/stan/cmdstan/stan/doc/api/html/check__ordered_8hpp_source.html]

Stan: src/stan/math/error\_handling/matrix/check\_ordered.hpp Source File


|  |  |
| --- | --- |
|  | Stan  2.5.0  probability, sampling & optimization |


- Main Page
- Related Pages
- Namespaces
- Classes
- Files

- File List
- File Members

All Classes Namespaces Files Functions Variables Typedefs Enumerations Enumerator Friends Macros Pages

- src
- stan
- math
- error\_handling
- matrix

check\_ordered.hpp

Go to the documentation of this file.

1 #ifndef STAN\_\_MATH\_\_ERROR\_HANDLING\_\_MATRIX\_\_CHECK\_ORDERED\_HPP

2 #define STAN\_\_MATH\_\_ERROR\_HANDLING\_\_MATRIX\_\_CHECK\_ORDERED\_HPP

3

4 #include <sstream>

5

6 #include <stan/math/error\_handling/dom\_err.hpp>

7 #include <stan/math/matrix/Eigen.hpp>

8 #include <stan/math/matrix/meta/index\_type.hpp>

9 #include <stan/meta/traits.hpp>

10

11 namespace stan {

12  namespace math {

13

28  template <typename T\_y, typename T\_result>

29  bool check\_ordered(const char\* function,

30  const Eigen::Matrix<T\_y,Eigen::Dynamic,1>& y,

31  const char\* name,

32  T\_result\* result) {

33  using Eigen::Dynamic;

34  using Eigen::Matrix;

35  typedef typename index\_type<Matrix<T\_y,Dynamic,1> >::type size\_t;

36

37  if (y.size() == 0) {

38  return true;

39  }

40  for (size\_t n = 1; n < y.size(); n++) {

41  if (!(y[n] > y[n-1])) {

42  std::ostringstream stream;

43  stream << " is not a valid ordered vector."

44  << " The element at " << stan::error\_index::value + n

45  << " is %1%, but should be greater than the previous element, "

46  << y[n-1];

47  std::string msg(stream.str());

48  return dom\_err(function,y[n],name,

49  msg.c\_str(),"",

50  result);

51  return false;

52  }

53  }

54  return true;

55  }

56  template <typename T\_y, typename T\_result>

57  bool check\_ordered(const char\* function,

58  const std::vector<T\_y>& y,

59  const char\* name,

60  T\_result\* result) {

61  if (y.size() == 0) {

62  return true;

63  }

64  for (int n = 1; n < y.size(); n++) {

65  if (!(y[n] > y[n-1])) {

66  std::ostringstream stream;

67  stream << " is not a valid ordered vector."

68  << " The element at " << stan::error\_index::value + n

69  << " is %1%, but should be greater than the previous element, "

70  << y[n-1];

71  std::string msg(stream.str());

72  return dom\_err(function,y[n],name,

73  msg.c\_str(),"",

74  result);

75  return false;

76  }

77  }

78  return true;

79  }

80  template <typename T>

81  bool check\_ordered(const char\* function,

82  const Eigen::Matrix<T,Eigen::Dynamic,1>& y,

83  const char\* name,

84  T\* result = 0) {

85  return check\_ordered<T,T>(function,y,name,result);

86  }

87  template <typename T>

88  bool check\_ordered(const char\* function,

89  const std::vector<T>& y,

90  const char\* name,

91  T\* result = 0) {

92  return check\_ordered<T,T>(function,y,name,result);

93  }

94

95  }

96 }

97 #endif

dom\_err.hpp

stan::math::check\_ordered

bool check\_ordered(const char \*function, const Eigen::Matrix< T\_y, Eigen::Dynamic, 1 > &y, const char \*name, T\_result \*result)

Return true if the specified vector is sorted into increasing order.

**Definition:** check\_ordered.hpp:29

stan::math::dom\_err

bool dom\_err(const char \*function, const T &y, const char \*name, const char \*error\_msg, const T\_msg error\_msg2, T\_result \*result)

**Definition:** dom\_err.hpp:36

traits.hpp

stan::math::index\_type

Primary template class for the metaprogram to compute the index type of a container.

**Definition:** index\_type.hpp:21

stan::error\_index::value

**Definition:** traits.hpp:20

index\_type.hpp

Eigen.hpp


---

[ Stan Home Page ]

*© 2011–2014,
Stan Development Team.*
